# Supplementary figures and images for: Blood-brain barrier-restricted translocation of Toxoplasma gondii from cortical capillaries
Source: eLife. 2021 Dec 8;10:e69182. doi: 10.7554/eLife.69182 (PMC8700292; doi:10.7554/eLife.69182)

Representative blot used in Figure 9a.

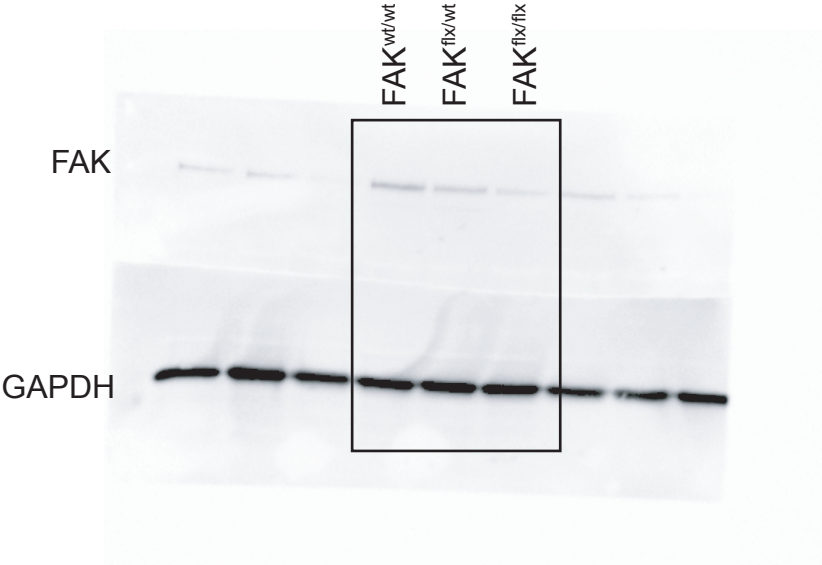

Blots used for bar graph in Figure 9a.

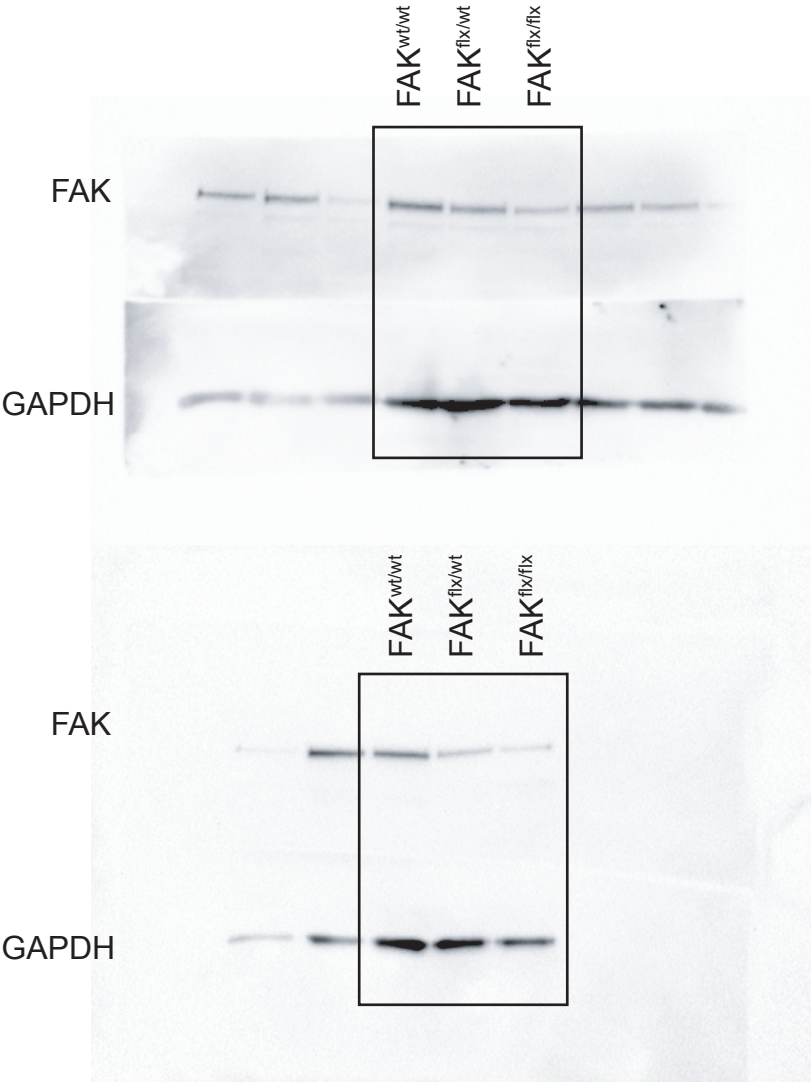

Supplement: Figure 9—source data 1. [file elife-69182-fig9-data1.zip › blots eLife.pdf]

Ladder  
flx/flx  
flx/wt  
flx/flx  
flx/wt  
flx/flx  
flx/wt

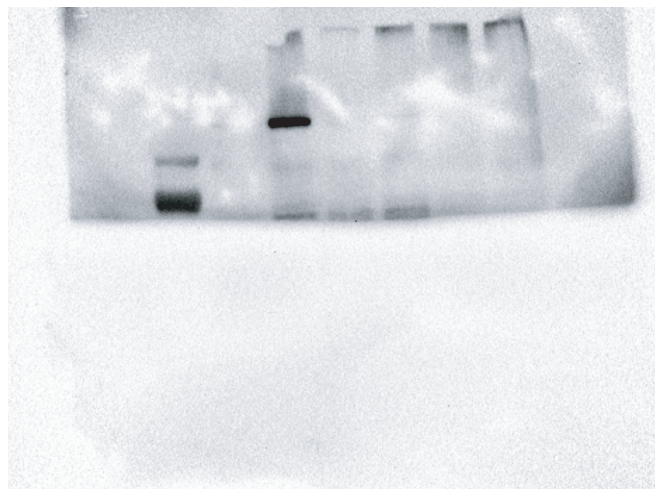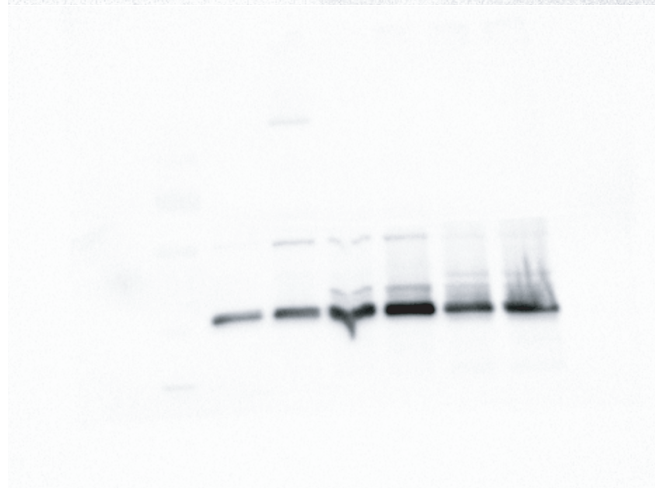

flx/flx  
flx/wt  
flx/flx  
flx/wt

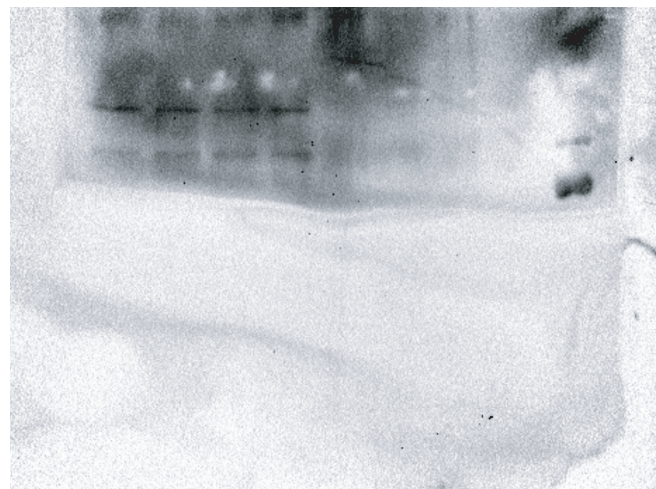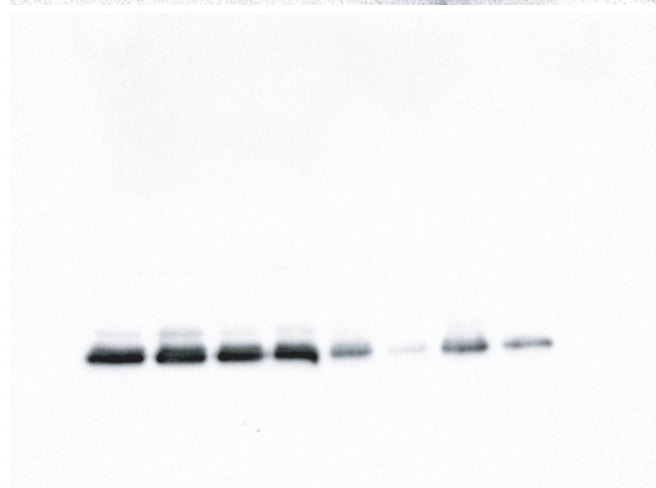

Ladder

Supplement: Figure 9—figure supplement 1—source data 1. [file elife-69182-fig9-figsupp1-data1.pdf]
